# Effects of Interferons and Viruses on Metabolism

**DOI:** 10.3389/fimmu.2016.00630

**Published:** 2016-12-21

**Authors:** Stephanie Deborah Fritsch, Thomas Weichhart

**Affiliations:** ^1^Institute of Medical Genetics, Medical University of Vienna, Vienna, Austria

**Keywords:** glycolysis, oxidative phosphorylation, fatty acid synthesis, fatty acid oxidation, cholesterol synthesis, mTOR, immunometabolism

## Abstract

Interferons (IFNs) are potent pleiotropic cytokines that broadly alter cellular functions in response to viral and other infections. These alterations include changes in protein synthesis, proliferation, membrane composition, and the nutritional microenvironment. Recent evidence suggests that antiviral responses are supported by an IFN-induced rewiring of the cellular metabolism. In this review, we discuss the roles of type I and type II IFNs in regulating the cellular metabolism and biosynthetic reactions. Furthermore, we give an overview of how viruses themselves affect these metabolic activities to promote their replication. In addition, we focus on the lipid as well as amino acid metabolisms, through which IFNs exert potent antiviral and immunomodulatory activities. Conversely, the expression of IFNs is controlled by the nutrient sensor mammalian target of rapamycin or by direct reprograming of lipid metabolic pathways. These findings establish a mutual relationship between IFN production and metabolic core processes.

## Introduction

Type I and II interferons (IFNs) are important cytokines that are induced upon viral infections (1). They promote a so-called “antiviral state” that limits viral replication in infected cells and viral spreading in non-infected cells. Additionally, IFNs are expressed during bacterial infections or autoimmune diseases and exert potent immunomodulatory functions. The human type I IFN family consists of 13 IFNα subtypes (14 in mice), one single IFNβ gene, and some further poorly analyzed genes (2). The sole representative of class II IFN is IFNγ, which is mainly produced by T cells and NK cells (2). IFNγ generally activates innate responses by augmenting inflammatory cytokine and chemokine production, microbial killing, and antigen presentation of macrophages and dendritic cells (3). Upon stimulation of extra- and intracellular pattern recognition receptors (PRR), including Toll-like receptors (TLR), nucleotide-binding oligomerization domain-like receptors, and retinoic acid-inducible gene I-like receptors, many immune cells, but also non-hematopoietic cells, are capable of inducing type I IFNs by a concerted activation of transcription factors called IFN-regulatory factors (IRFs) (4). Expression of IFNs is also dependent on the sensing of the extra- and intracellular microenvironment by the mammalian target of rapamycin (mTOR) network (5). mTOR complex 1 (mTORC1) integrates the main classes of nutrients and energy sources [amino acids, glucose, lipids, and adenosine triphosphate (ATP)] to couple the environmental status with cellular activation and translation (5). Activation of mTORC1 is required to induce the translation as well as the activation of IRFs, including IRF5 and IRF7, to maximize type I IFN production (6–9). IFNα and IFNβ bind a heterodimeric membrane receptor consisting of the interferon alpha and beta receptor subunit 1 (IFNAR1) and IFNAR2 (10). Receptor engagement activates the receptor-associated protein tyrosine kinases Janus kinase 1 (JAK1) and tyrosine kinase 2 (TYK2), which phosphorylate and activate the transcription factors signal transducer and activator of transcription 1 (STAT1) and STAT2 (10). In contrast, the dimeric IFNγ receptor consists of the interferon gamma receptor 1 (IFNGR1) and IFNGR2 and activates the receptor-associated tyrosine kinases, JAK1 and JAK2, which solely activate STAT1 (11).

Type I IFNs and IFNγ induce the transcriptional upregulation of several hundred interferon stimulated genes (ISGs) (1, 4). Three families of ISGs have been extensively studied with respect to their antiviral activities. These genes encode the double-stranded RNA-activated protein kinase (PKR), the 2′,5′-oligoadenylate synthetases (OAS), and the Mx protein(s) (1, 12). They actively participate in inhibiting viral replication by different mechanisms. PKR is an IFN-inducible and RNA-dependent kinase that phosphorylates the translation initiation factor 2α (eIF2α), which inhibits cellular and viral translation (13). Activation of OAS by binding of dsRNA stimulates RNase L activity, which cleaves cellular and viral ssRNA to inhibit protein expression (13). Mx proteins are GTPases that often associate with nucleocapsid-like viral structures to trap and inhibit viral replication (14).

This review focuses on additional roles of IFNs involving the regulation of the cellular metabolism. The following sections discuss recent evidence and older observations of how type I and II IFNs modulate metabolic pathways to generate an antiviral state and influence subsequent immune responses.

## Cellular Metabolism

The principal purpose of metabolism is the conversion of nutrients to energy to maintain all cellular processes and the delivery of building blocks for the biosynthesis of proteins, lipids, nucleic acids, and some carbohydrates. Viruses are incapable of metabolizing on their own and are, therefore, completely dependent upon host metabolism. Their life cycle requires an energy-demanding synthesis of high levels of proteins, glycoproteins, nucleic acids, and sometimes lipids. Therefore, there is a mutual relationship between viral replication, metabolism, and host defense. First, we will discuss the basic principles of metabolism. Afterward, we will continue to elaborate on specific pathways of metabolism that are affected by IFNs or viral infection.

### Energy Metabolism

**Figure 1:**
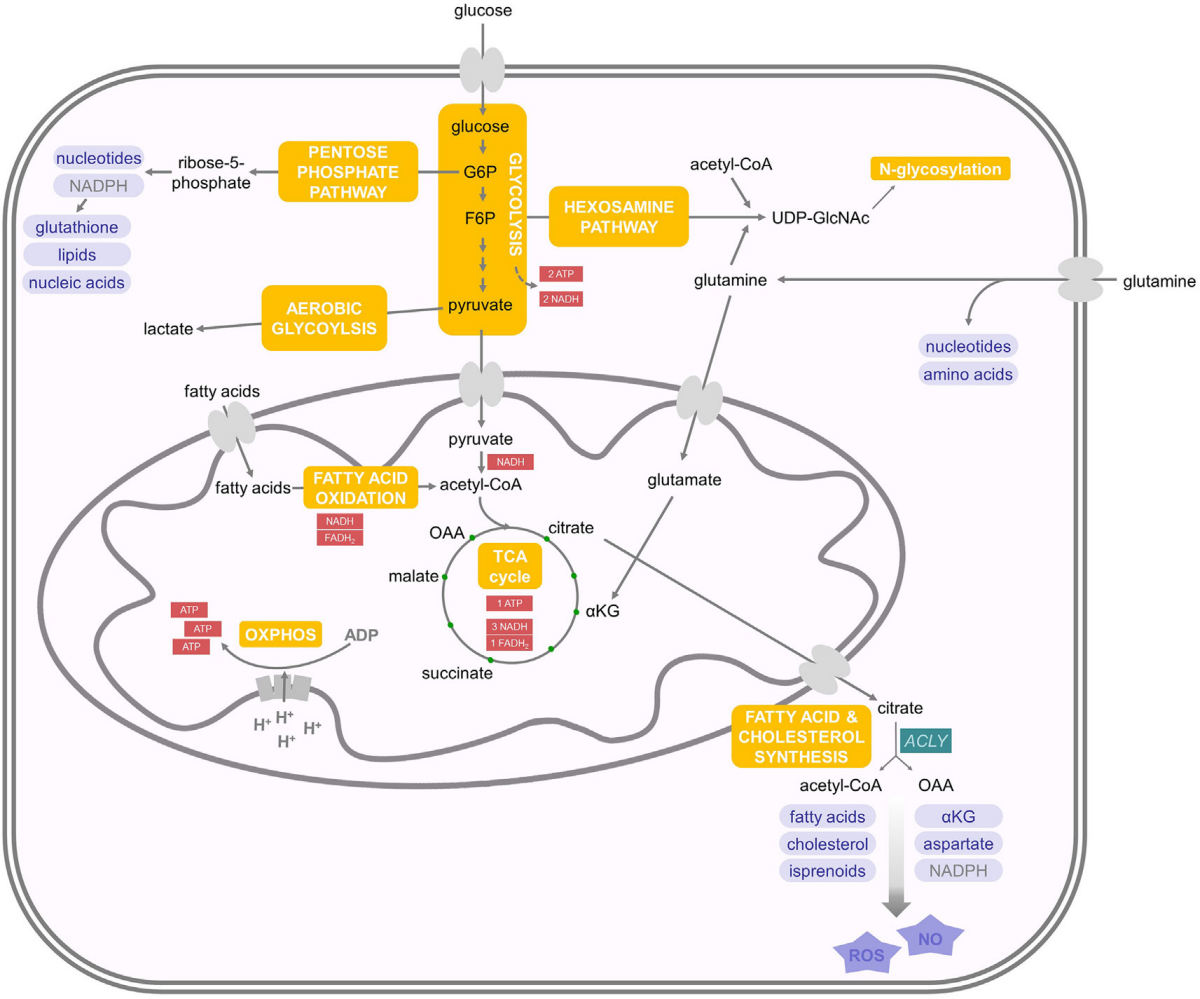
**Energy and biosynthetic metabolism**. Glucose is taken up and metabolized in the cytoplasm to pyruvate in a process called glycolysis. Pyruvate is then transported into the mitochondria and oxidized into acetyl coenzyme A (acetyl-CoA), which enters the tricarboxylic acid (TCA) cycle. The molecules NADH and FADH_2_ produced until this point are the inputs for the electron transport chain. Another important energy source are fatty acids, whose oxidation delivers acetyl-coA. Aerobic glycolysis takes place in proliferating (and cancer) cells and describes the phenomenon of increased glucose uptake and glycolysis with the subsequent production of lactate. Glutamine is another carbon source that can be transformed to αKG and, therefore, enters the TCA cycle. Glutamine can also be used as nitrogen donor in the hexosamine pathway, which requires F6P and is important for N-glycosylation of proteins. G6P can feed into the pentose phosphate pathway, which is important for the production of nucleotides and NADPH. Mitochondrial citrate can enter the cytoplasm and feeds into *de novo* fatty acid synthesis. For further details consult the text.

The central nutrients, used by eukaryotic cells to generate energy in the form of ATP, are carbohydrates, amino acids, and fatty acids (FAs) (15). In the presence of oxygen, non-proliferating cells take up the carbohydrate glucose and metabolize it in the cytoplasm to pyruvate through a process called glycolysis (Figure 1). This results in a net production of two ATPs and the reduction of two nicotinamide adenine dinucleotide (NAD) molecules to NADH. Pyruvate can be transported into the mitochondria, where it is oxidized into acetyl coenzyme A (acetyl-CoA) with the production of one molecule of carbon dioxide and one more NADH. Acetyl-CoA acts as fuel for the tricarboxylic acid (TCA) cycle (also known as citric acid or Krebs cycle), through which it is completely oxidized to carbon dioxide with the net production of three molecules of NADH, one molecule of ATP (or guanosine triphosphate GTP), and one molecule of the reduced form of flavin adenine dinucleotide (FADH_2_) (15). The molecules of NADH and FADH_2_, generated until this point, are the inputs for the electron transport chain. They are used to establish a proton gradient at the inner mitochondrial membrane, which finally generates ATP from adenosine diphosphate in a process called oxidative phosphorylation (16) (Figure 1). In summary, from one molecule of glucose, theoretically, 36 equivalents of ATP can be generated in eukaryotes, although due to proton leakage and inefficiencies of the ATPase, the observed yield is about 30 ATPs (17). Importantly, glucose is not the only energy source, which can be used by eukaryotic cells. The amino acid glutamine is a second carbon source that can be converted to α-ketoglutarate (αKG) as oxidative substrate to fuel the TCA cycle (16, 18). Moreover, fatty acid oxidation (FAO) in the mitochondria generates acetyl-CoA, NADH, and FADH_2_, which are further used to generate ATP (19). FAs are the most energetic nutrients, yielding the highest levels of ATP on an energy per gram basis. Hence, glycolysis and the TCA cycle are the central cellular respiratory systems of eukaryotic cells (15).

### Anabolic Metabolism

When cells start to proliferate, there is increasing demand of nutrients for energy production as well as biosynthesis of novel molecules (20). Therefore, proliferating cells increase glucose uptake and glycolysis, but do not oxidize all of the additional glucose-derived pyruvate in the TCA cycle. Instead, the pyruvate is reduced to lactate despite the presence of oxygen, which is therefore called aerobic glycolysis (21) (Figure 1). This effect was first described in tumor cells by Otto Warburg and is now called the Warburg effect (22). It is important to note that TCA flux is reduced but maintained during aerobic glycolysis in proliferating cells. Although aerobic glycolysis generates only two molecules of ATP, it is thought to generate cellular building blocks for rapidly proliferating cells (15). However, also amino acids are important contributors to increased cell mass in proliferating cells (23). Many glycolytic intermediates provide backbone carbons for multiple non-essential amino acids or function as substrates for the biosynthesis of phospholipids and triacylglycerols. In addition, the glycolytic intermediate glucose-6-phosphate (G6P) can feed into the pentose phosphate pathway to generate ribose-5-phosphate, which is important for nucleotide biosynthesis as well as the conversion of nicotinamide adenine dinucleotide phosphate to its reduced form NADPH (Figure 1). NADPH is used as reducing agent in lipid or nucleic acid synthesis and protects against cellular oxidative stress by generating reduced glutathione that inactivates reactive oxygen species (ROS) (e.g., H_2_O_2_) and free radicals (16). αKG, derived from glutamine, can be metabolized to malate and then to pyruvate to support NADPH generation in a process called glutaminolysis (16). Furthermore, glutamine is used as nitrogen donor for the biosynthesis of nucleotides, non-essential amino acids, and hexosamines. The hexosamine pathway requires fructose-6-phosphate (F6P) from glycolysis and acetyl-CoA, in addition to glutamine to produce uridine diphosphate-*N*-acetylglucosamine (UDP-GlcNAc), which is important for N-glycosylation of proteins (24). Therefore, N-glycosylation represents a nutrient-sensitive protein modification, which regulates the glycosylation of IFNs and viral glycoproteins (25). This modification is involved in protein trafficking, in viral entry, and in evading the immune system’s detection by some viruses (26).

As described above, during aerobic glycolysis, the TCA cycle is sustained in proliferating cells by glucose-derived pyruvate, as well as by replenishing depleted intermediates in the form of, e.g., glutamine in a process called anaplerosis (27, 28). The TCA cycle contributes many intermediates that act as biosynthetic substrates. For example, mitochondrial citrate can feed into *de novo* FA and cholesterol synthesis upon its export to the cytoplasm, where it is converted to acetyl-CoA and oxaloacetate by ATP citrate lyase (ACLY). Cytoplasmic acetyl-CoA is then the substrate for FAs, cholesterol, and isoprenoid synthesis (Figure 1). Phospholipids are generated from FAs and, together with cholesterol, form the majority of the lipid bilayers of the cellular membranes. Oxaloacetate is further metabolized to yield αKG and NADPH (29). Alternatively, oxaloacetate can be transaminated to aspartate, which acts as a carbon source in nucleotide biosynthesis. In addition, ACLY-derived acetyl-CoA and oxaloacetate can serve as precursors for nitric oxide (NO) and ROS production (30, 31).

## Effects of IFNs on Energy Metabolism

While it has been known for a long time that viral infections and IFNs interfere with lipid metabolism including FA and cholesterol synthesis (described below), recent studies have shown a more general influence of IFNs on the energy metabolism of cells.

**Figure 2:**
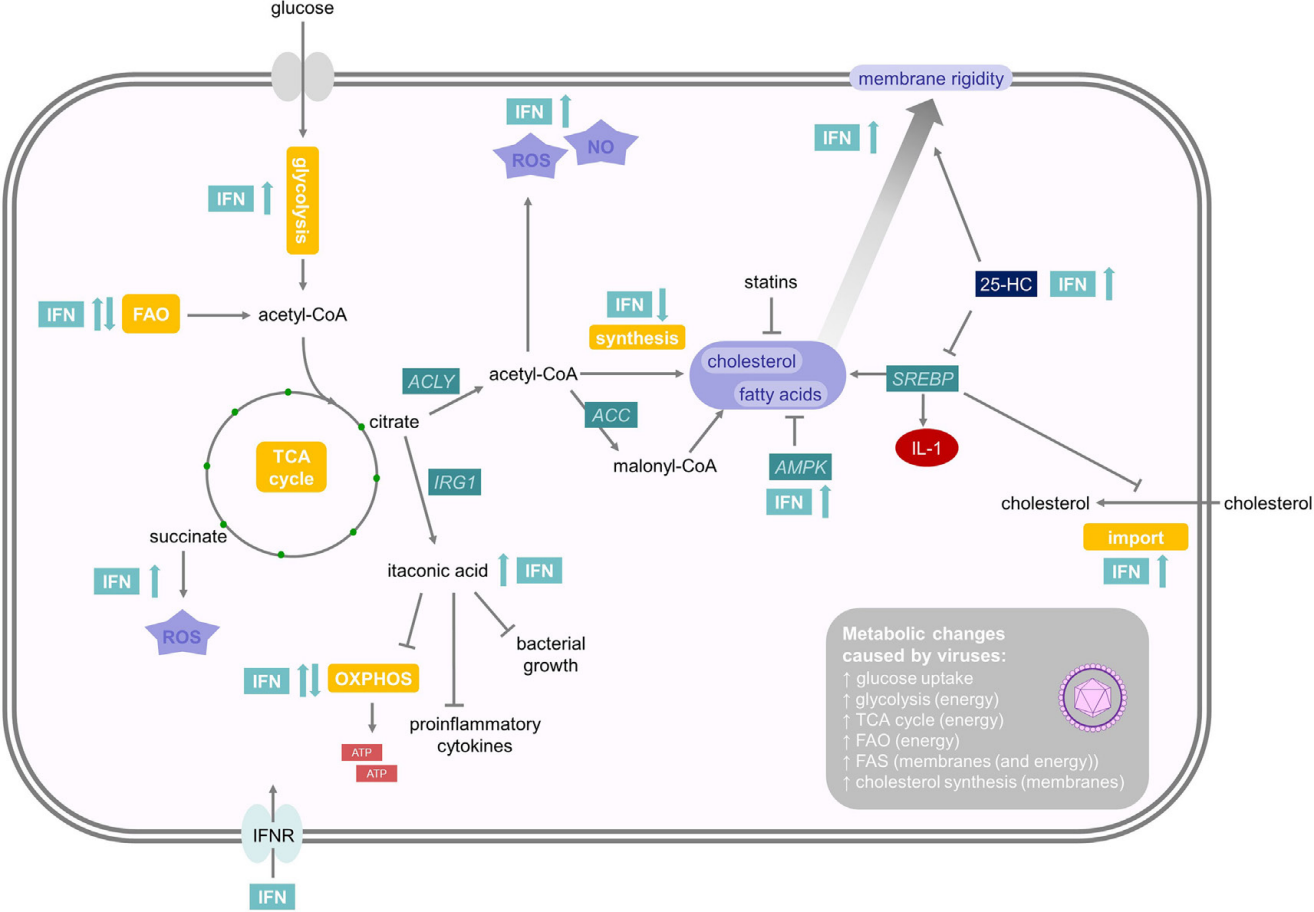
**Effects of interferons (IFNs) on energy and lipid metabolism**. Type I IFNs promote glycolysis while mitochondrial respiration is regulated cell-type specifically. Citrate can induce the formation of itaconic acid or acetyl coenzyme A (acetyl-CoA). Itaconic acid is a bactericidal metabolite, which inhibits proinflammatory cytokine expression and mitochondrial respiration. Acetyl-CoA can either promote NO and reactive oxygen species (ROS) production or initiate fatty acid (FA) and cholesterol synthesis. As viral replication is an energy-demanding process, which depends on protein and nucleotide synthesis, most viral infections enhance FA and cholesterol synthesis, which, on the other hand, can be reduced by adenosine monophosphate-activated protein kinase and statins. 25-HC is a soluble antiviral factor that broadly inhibits growth of many enveloped viruses by inhibiting sterol regulatory binding protein and enhances membrane rigidity. IFNs also promote subsequent NO and ROS production. For further details consult the text.

Generally, a theme emerges that type I IFNs promote glycolysis (Figure 2). For example, IFNβ stimulates a PI3K/AKT-dependent glucose uptake in mouse embryonic fibroblasts that may enhance ATP production (32). Inhibition of IFNβ-induced glycolysis with 2-deoxyglucose (2-DG), a competitive inhibitor of hexokinase, the first enzyme in the glycolysis cascade, enhances replication of coxsackievirus B3 *in vitro* (32). This suggests that enhanced glycolysis may support the establishment of an antiviral state. Similarly, injection of the synthetic dsRNA poly(I:C), a TLR3 and melanoma differentiation antigen 5 agonist, into mice induces an increase in glycolysis in splenic CD11c^+^ MHCII^+^ DCs *ex vivo* (33). This increase is dependent on IFNAR1 and thus mediated by type I IFNs. Increased glycolysis often is accompanied by a decreased oxidative consumption, and this Warburg effect depends on expression of hypoxia-inducible factor 1α (Hif1α) and is required to efficiently prime CD8^+^ and CD4^+^ T cells *in vivo* (33). In macrophages, TYK2 and IFNAR1 are also required for an increase in glycolysis-mediated lactate production (34). IRF5 increases glycolysis in macrophages through a glycolytic gene expression induced by activation of AKT2 (35). In a human squamous carcinoma cell line, expression of type I IFN-regulated STAT1 promotes aerobic glycolysis and decreases oxidative phosphorylation, which contributes to tumor growth in a xenograft model (36). Other studies also showed that mitochondrial respiration and ATP production are diminished upon type I IFN treatment in mouse L929 or human Daudi cells (37). In humans, IFNβ-treated multiple sclerosis patients exhibited a dose-dependent reduction of ATP levels in isolated CD4^+^ T cells (38).

However, there are also important examples, where type I IFN stimulates oxygen consumption. Plasmacytoid dendritic cells (pDCs) are specialized immune cells devoted to the production of large amounts of type I IFNs after viral recognition (39). Mouse pDCs upregulate oxidative phosphorylation and ATP production 24 h after stimulation with poly(I:C) or directly after type I IFN treatment through an autocrine loop (40). This boost in energy production is required for full immune effector functions *in vitro* and for fighting lymphocytic choriomeningitis virus (LCMV) infection *in vivo*. This increase in oxidative phosphorylation and mitochondrial respiration is fueled by FAO (40). Interestingly, the FAs required for FAO seem to be a result of *de novo* fatty acid synthesis (FAS) from glycolysis-derived pyruvate. The stimulating effect of type I IFN on increased oxygen consumption was also observed on conventional DCs, keratinocytes, or memory T cells, but not on effector T cells (40). This increase in ATP and mitochondrial fitness may support the energetic demands of high cytokine production in pDCs and in non-hematopoietic cells to support survival during viral infection. In contrast, stimulation of human pDCs with influenza virus induced a Warburg-like remodeling of the energy metabolism, including enhanced glycolytic flux and decreased mitochondrial respiration (41). These studies in total suggest that type I IFN, by canonical pathway activation through IFNAR1, Tyk2, and STAT1, mediates an induction of glycolysis, whereas mitochondrial respiration seems to be regulated cell-type specifically (Figure 2). Interestingly, type I IFN and IFNγ induce lipolysis in cultured adipocytes and in mice *in vivo* and may thus supply cells with FAs (42). However, this function of IFN has not been thoroughly investigated.

A decrease in oxidative phosphorylation reduces mitochondrial ATP production, which may still be compensated by ATP produced through aerobic glycolysis, whose flux can be dramatically increased when glucose is not limited (43). Reduced mitochondrial respiration frees TCA intermediates, which can be used in subsequent biosynthetic reactions. For example, activation of macrophages with IFNγ and lipopolysaccharide (LPS) induces high levels of glycolysis and a break of TCA flux. This leads to the accumulation of succinate and citrate in conjunction with induction of FAS (44). Succinate can drive mitochondrial ROS production (45), which is a conserved response against many pathogens (46) but may also cause tissue pathology (as discussed below) (47). Naujoks et al. showed that type I and II IFNs control *Legionella pneumophila* infection in alveolar macrophages by induction of a bactericidal molecule (48). In fact, *Legionella*-infected macrophages induce IFN-dependent expression of immune-responsive gene (IRG) 1 that mediates production of itaconic acid (also known as methylenesuccinic acid). This molecule is bactericidal against a number of extracellular multidrug-resistant, Gram-positive, and Gram-negative bacteria (48). Itaconic acid is produced by IRG1 through decarboxylation of cis-aconitate, a TCA intermediate that is formed from citrate (49, 50) (Figure 2). Except of being a bactericidal metabolite, itaconic acid also inhibits proinflammatory cytokine expression (51) and mitochondrial respiration (51, 52). Stimulation of macrophages with poly(I:C), IFNγ, or LPS can also increase the expression and activation of ACLY (31, 34). This, in turn, enhances the conversion of citrate into acetyl-CoA and oxaloacetate, which promotes subsequent NO and ROS production.

## Modulation of Lipid Synthesis by Viruses and IFNs

Viral replication depends on a massive induction of protein and nucleotide synthesis. Therefore, most viruses themselves upregulate carbon fluxes and promote efflux to nucleotide and amino acid biosynthesis (53). Additionally, virus entry, replication, and assembly rely on membranous networks, surrounding and residing within the host cells. These include the plasma, the endolysosomal, and the endoplasmic reticulum (ER) membranes (54–56), which all function as scaffolds to recruit and concentrate viral and host components, necessary for viral replication and assembly (57). Many viruses induce changes in membrane fluidity and a massive proliferation of membranes such as the ER, which is the place for translation of secretory and membrane proteins and for N-glycosylation (54, 55). Obviously, enveloped viruses need not only to induce membrane generation but also alter the composition of the cell membrane to meet their needs for effective infectious progeny particles (58). However, viral replication is also a highly energy-demanding process; therefore, utilizing all available energy to produce ATP is rate-limiting for some viruses (58).

### FA Synthesis and IFNs

In light of these functional prerequisites, it comes as no surprise that most viral infections enhance FA and cholesterol synthesis to support generation of membranes and ATP production (53) (Figure 2). For example, human cytomegalovirus (HCMV) upregulates most metabolic pathways in infected fibroblasts and drives flux from glycolysis through the TCA cycle to FAS (59). Inhibition of FAS suppresses replication of HCMV (59). Mechanisms of HCMV-induced metabolic reprograming include the activation of the glucose transporter Glut4 and inductions of ACLY and acetyl coenzyme A carboxylase (ACC) (53, 60). After ACLY-dependent generation of acetyl-CoA in the cytoplasm, ACC carboxylates acetyl-CoA to malonyl-CoA, which is a critical rate-limiting step in FAS (19). ACLY and ACC are currently evaluated as therapeutic targets for cancer, obesity, diabetes, and viral infections (61, 62). Similarly, influenza A, flaviviridae family members including hepatitis C virus (HCV) and West Nile virus (WNV), enteroviruses including poliovirus and coxsackievirus B3, rotavirus, rift valley fever virus, and respiratory syncytial virus depend on FAS for viral replication, making its modulation an attractive therapeutic target (63–65). 5′-adenosine monophosphate-activated protein kinase gets activated after certain virus infections, such as rift valley fever virus or coxsackievirus B3, and potently inhibits FAS (66, 67). Some viruses, such as influenza A, use FAS to induce the production of prostaglandin E2, which inhibits IFN expression and promotes apoptosis in macrophages (68). Viral replication is a highly energy-demanding process. Therefore, utilizing all available energy is critical and rate-limiting for some viral infections. Hence, some viruses depend on FAS and their degradation by FAO to produce ATP. In this respect, Vaccinia virion assembly is dependent on ATP synthesis fueled by FAS and FAO (69). Dengue virus, on the other hand, induces FAO by an autophagy-dependent processing of lipid droplets and triglycerides to generate ATP for efficient replication (70). Nevertheless, dengue virus also induces FAS to support virus replication (71). Hence, channeling the FAs from biosynthesis to catabolism by the induction of FAO, as seen in pDCs (40), could represent a novel powerful antiviral mechanism of IFN. However, further work is required to elucidate whether this represents a general antiviral mechanism.

### Cholesterol Homeostasis and IFN Responses

Many viruses do not only modulate FAs, but also cholesterol homeostasis to enhance their replication efficiency. For example, WNV upregulates biosynthesis of cholesterol, redistributing it to viral membranes in the phase of replication (72). Moreover, HCV, hepatitis B virus (HBV), measles, human immunodeficiency virus (HIV), and dengue virus also change cholesterol pathway gene expression in a variety of cellular systems (73–76). Pharmacological disruption of cholesterol synthesis, e.g., by statins, often results in the inhibition of viral replication (77–82). Recent evidence has shown that an important antiviral mechanism of type I IFN seems to be inhibition of cholesterol and fatty acid biosynthesis derived from glucose (83, 84). Type I IFN reduces cholesterol synthesis upon CMV, herpes simplex (HSV1), semliki forest virus, vaccinia virus (VV), and adenovirus (ADV) infection in bone marrow-derived macrophages, which is dependent on IFNAR1 and TYK2 (83). Similarly, infection with murine gammaherpesvirus-68 (MHV-68) reduces cholesterol and long chain FAS in macrophages (84). It is important to note that total cholesterol levels are not strongly affected upon inhibition of cholesterol synthesis by IFN due to an enhancement of cholesterol import (84) (Figure 2). Similarly, WNV infection enhances cholesterol synthesis, but total cholesterol levels do not change (72). Inhibition of cholesterol biosynthesis has a direct antiviral effect. The sterol regulatory binding protein 2 (SREBP2), together with SREBP1, are the main transcription factors involved in coordinating the regulation of the sterol biosynthesis pathway (85). IFNs potently inhibit the transcription and expression of SREBP2 *via* IFNAR1 (83). On the other hand, WNV-induced redistribution of cellular cholesterol downregulates the IFN-stimulated JAK–STAT antiviral signaling response to infection potentially by influencing lipid raft signaling (72).

Fascinatingly, limiting cholesterol synthesis alone induces spontaneous type I IFN production and enhances antiviral immunity (84). Deletion of SREBP2 or the ER chaperone SCAP, which regulates SREBP2, reduces synthesis but enhances the uptake of cholesterol. This shift induces spontaneous IFN signaling that is strongly enhanced upon viral infection in bone marrow-derived macrophages or mouse embryonic fibroblasts. The IFN response in these cells is dependent on the cGAS–STING–TBK1–IRF3 pathway (84). The stimulator of interferon gene (STING) protein is anchored on the ER and Golgi apparatus, suggesting that cholesterol levels and potentially lipid rafts in these membranes may modulate STING signaling. Moreover, it is interesting to note that cyclic guanosine monophosphate-adenosine monophosphate synthase (cGAS), which senses DNA from viral infections, is evolutionarily conserved with OAS, another important antiviral protein that is induced by IFNs (86). In summary, many viruses increase cholesterol and FAS. This is counteracted by type I IFN signaling, which limits FA and cholesterol synthesis. Reducing cholesterol synthesis alone induces IFN production establishing an inflammatory circuit, which links the regulation of the sterol pathway with the antiviral IFN defense responses. However, one report noted enhanced cholesterol synthesis derived from acetate in IFNβ-treated HeLa cells (87).

### Regulation of Membrane Function by 25-Hydroxycholesterol

Cholesterol-derived metabolites such as oxysterols are important systemic mediators that regulate many immunological functions (88). IFNs or viral infections lead to the induction and secretion of one oxysterol in macrophages: 25-hydroxycholesterol (25-HC) (89) (Figure 2). 25-HC is a soluble antiviral factor, generated from cholesterol by IFN-dependent activation of cholesterol-25-hydroxylase *via* STAT1 (89). 25-HC broadly inhibits growth of many enveloped viruses, such as vesicular stomatitis virus, HSV, HIV, MHV68, and Ebola virus, by suppressing membrane fusion between the virus and the host cell (90, 91). Mechanistically, 25-HC seems to incorporate into the membrane and/or modify the membrane composition (91). Indeed, an IFN-dependent increase in plasma membrane rigidity has long been observed in several previous studies (92–94). IFNβ augments membrane rigidity already after 30 min, and this is maintained for 2 days making it a powerful antiviral mechanism to prevent viral infection and spreading (93, 95). Nevertheless, type I IFNs decrease membrane contents of saturated FAs and increase unsaturated FAs (92). In patients with chronic hepatitis C infection, treatment with IFNα2 reduces the deformability and membrane fluidity of red blood cells, which may result in hemolytic anemia, a frequent side effect of IFN therapy (96). Another downside of IFN-induced 25-HC expression is its capacity to inhibit SREBP1, which not only drives FAS but also stimulates transcription of interleukin-1, a secreted inflammatory protein with wide-ranging antibacterial functions (97). This may explain why IFNs, produced during viral infections, enhance the subsequent susceptibility to bacterial or fungal infections (98–100). Generally, there is increasing awareness of a close relationship between membrane lipid dynamics and innate immune responses (101).

## Amino Acid Metabolism and IFNs

### IFNs Deplete Polyamines to Restrict Virus Replication

**Figure 3:**
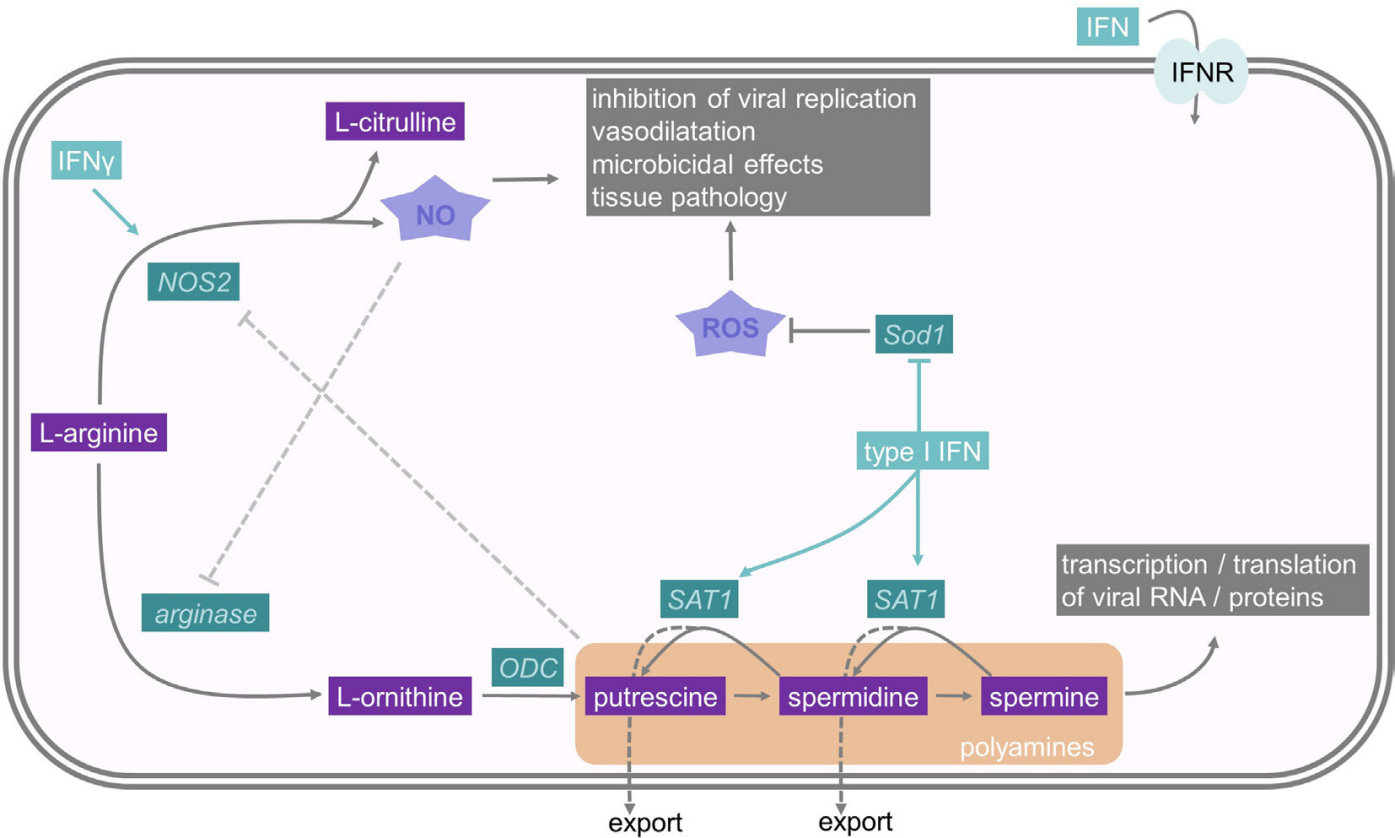
**Interferons and their influence on nitric oxide and polyamine metabolism**. The polyamines putrescine, spermidine, and spermine derive from the amino acid l-arginine. One rate-limiting enzyme in polyamine synthesis is ODC, while Spermidine–spermine acetyltransferase (SAT1) is an important enzyme in polyamine catabolism. As polyamines are important for viral replication, SAT1 constitutes an important interferon stimulated gene. NO also derives from l-arginine, and therefore, depletes the substrate for PA synthesis. It has microbicidal effects and reduces viral replication. Sod1 is an antioxidative molecule which resolves oxidative stress. For further details consult the text.

Polyamines are a family of small polycationic molecules, derived from decarboxylation of the amino acid ornithine, that classically comprise three molecules: putrescine, spermidine, and spermine (102). Spermine is generated from spermidine, which itself is produced from putrescine (Figure 3). Ornithine, which generates putrescine, is produced from l-arginine by arginase (103). Polyamines bind DNA, RNA, and proteins and are implicated in supporting transcription, translation, and deacetylation to influence a plethora of different cellular functions, including proliferation, apoptosis, autophagy, and gene regulation (103). Spermidine–spermine acetyltransferase (SAT1 or SSAT) acetylates spermidine and spermine, which promotes either their conversion back to putrescine or their export from the cell (102, 103). Interestingly, type I IFNs induce the expression of SAT1 and, therefore, deplete spermidine and spermine levels (104). The depletion of these two polyamines has a strong antiviral effect and inhibits replication of the RNA viruses, Zika virus and Chikungunya virus (104). Mechanistically, polyamines seem to be important for transcription and translation of viral RNA and proteins. Limiting polyamine synthesis, therefore, emerges as a novel antiviral strategy and SAT1 constitutes an important ISG.

### IFNs Stimulate Arginine-Dependent NO Production

NO is a gaseous and inorganic free radical best known for its vasodilatory and microbicidal effects (105). However, NO is also an important mediator in intracellular inhibition of viral replication, which results in lower viral yields and more efficient host clearance of the infection (106). NO is produced by the enzymatic modification of l-arginine to l-citrulline by NO synthases (NOS) (Figure 3). NOS type 2 (NOS2, iNOS) is an IFNγ-inducible protein in macrophages and requires IRF1 as a transcription factor, which itself is regulated by STAT1 (106, 107). Molecularly, the antiviral activities of NO are poorly described, but one demonstrated mechanism is nitrosylation of viral molecules (108). For example, NO S-nitrosylates the cysteine residue in the active site of Coxsackievirus protease 3C, thus inhibiting protease activity and interrupting the viral life cycle (109). In addition, the generation of NO by NOS2 depletes the common substrate l-arginine and, subsequently reduces polyamine levels, as described above. Moreover, this relieves a feedback inhibition mechanism, because polyamines can directly inhibit NOS2 (110). Hence, IFN-induced NOS2 and SAT1 induction have antiviral effects due to a coordinated shift from polyamine synthesis to NO production.

### Type I IFNs Promote Oxidative Stress and Tissue Damage

Viral or bacterial infections often cause immunopathology and tissue damage, not only because of the pathogens destroying the tissue but because of an overactivation of the immune system, which promotes tissue destruction. For example, excessive type I and II IFN production can drive tissue damage by proinflammatory actions on innate and adaptive immune cells, as well as the induction of apoptosis (111–113). IFNγ can cause the production of ROS, which induces apoptosis (114). NO contributes to tissue damage, especially if substantial numbers of IFN-activated macrophages produce large micromolar quantities of NO (115). First, NO can have proinflammatory effects on other cells of the immune system causing hyperactivation and immunopathology (105). Moreover, NO can rapidly react with hydrogen peroxide (H_2_O_2_) to form peroxynitrite (ONOO^−^), which nitrates proteins and is highly toxic, leading to the accumulation of injurious intracellular oxidants and to DNA damage (116). This NO-induced oxidative stress causes cytotoxicity, which promotes cellular and organ dysfunction (115). Currently, there is no clear-cut way of predicting whether NO has a more important role in viral clearance or in tissue pathology for a particular viral pathogen.

There are additional metabolic mechanisms explaining how IFN signaling can promote immunopathology. Infection of mice with LCMV causes a dysregulation of the redox system in the liver. In this infection model, the early production of type I IFN causes tissue pathology due to the downregulation of superoxide dismutase 1 (Sod1) in the liver (117). Sod1 is a ubiquitously expressed antioxidative molecule, which can protect cells from oxidative stress by scavenging ${\text{O}}_{2}^{-}$ radicals (118) (Figure 3). Hence, type I IFN-mediated oxidative stress may be a key mediator of virus-induced liver damage, and this suggests that early antioxidant treatment may be therapeutically helpful in ameliorating tissue damage. On the other hand, this oxidative stress, induced by the downregulation of Sod1, may also be part of an immediate antioxidant host defense system against pathogens (117).

### Depletion of Tryptophan as an Immunomodulatory Mechanism of IFN

**Figure 4:**
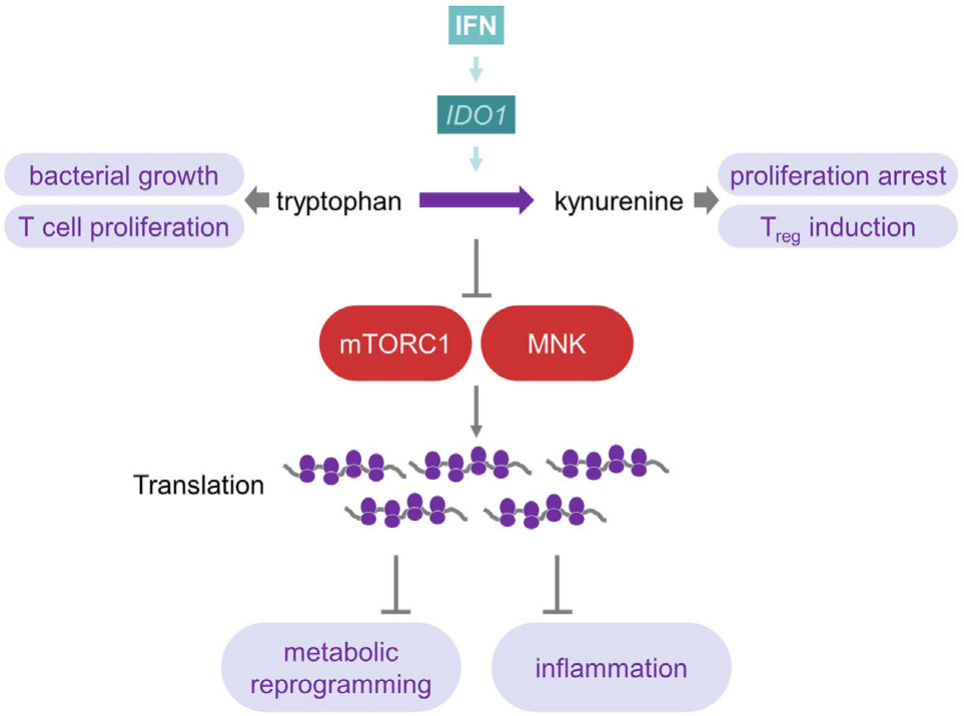
**Interferon influences tryptophan metabolism to reprogram metabolism and inflammation**. IDO1, which is induced by IFNs, catalyzes the production of Kyn from Trp. As many microorganisms rely on this amino acid, this represents a mechanism against bacterial infections. Furthermore, Trp is important for T-cells, and its depletion, therefore, inhibits T-cell effector immunity, while Kyn promotes T-cell tolerance by inducing T_regs_. Depletion of Trp causes inhibition of mTOR complex 1 as well as MAP kinase-interacting kinases and, therefore, induces changes at the translational level of metabolism and inflammation. For further details consult the text.

Indoleamine-2,3-dioxygenase 1 (IDO1) is an intracellular, non-secreted enzyme, which catalyzes the production of kynurenine (Kyn) derivatives from the essential amino acid tryptophan (Trp) (119). The IDO1 promoter region contains two IFN-stimulated response elements and three IFNγ-activated sites. Hence, IFNγ is the most potent inducer of IDO1 expression in many cells, including macrophages, fibroblasts, and pDCs (120, 121) (Figure 4). Although type I IFNs are able to directly induce IDO1, the maximum IDO1 expression requires co-stimulation with TNF-α or LPS (122, 123). Strong activation of IDO1 by IFNγ decreases serum levels of Trp. Since many microbial organisms rely on Trp, its degradation by IDO1-expressing cells of the innate immune system seems to be a major immune mechanism against bacterial infections (124). In fact, IFNγ-induced IDO1 expression seems to be protective in *Toxoplasma gondii, Salmonella enterica* serovar Typhi (*S*. Typhi), or *Chlamydia pneumoniae* infections (124, 125). For example, IFNγ-primed macrophages effectively contain intracellular replication of *S*. Typhi depending on the activation of IDO1 (125).

IDO1 expression also plays an important role during viral infections, such as HIV, influenza, Epstein–Barr, HBV, and HCV (124, 126). However, in viral infections, the induction of IDO1 by IFN seems to be generally harmful by the establishment of an immunotolerogenic microenvironment (124). Trp is important for activation and proliferation of T cells. Hence, Trp depletion inhibits T cell immunity and, moreover, the oxidation of Trp by IDO1 generates Kyn derivatives, which promote T cell tolerance by induction of regulatory T cells (121) (Figure 4). Therefore, mice lacking IDO1 exhibit significantly lower morbidity after sub-lethal influenza A infection by generating a stronger influenza-specific effector CD8 T cell response, though viral clearance rates are unaffected by IDO1 ablation (127). Similarly, genetic ablation of IDO1 or chemical inhibition with 1-methyl-d-l-tryptophan suppresses viral replication of murine leukemia virus *in vivo* and upregulates type I IFN production (128). In conclusion, in various chronic infections, autoimmune diseases, and cancer, an increased expression of IDO1, besides its antiviral effects, may promote an immunosuppressive environment, which potentially contributes to disease (119, 121, 123, 125).

### Tryptophan Depletion Suppresses mTOR-Mediated Translation and Modulates IFNγ-Dependent Metabolic Processes

The importance of translational control of many cellular responses, including metabolism, is increasingly appreciated (129, 130). On the one hand, type I IFNs cause massive translational inhibition as antiviral strategy (131), but on the other hand, they do promote the translation of ISGs, including PKR (10). Molecularly, AKT-mTORC1, mTORC2, and MAP kinase-interacting kinases (MNK), as well as eukaryotic initiation factor 4E (eIF4E) signaling are transiently activated by type I IFNs. An increase in ISG mRNA translation follows, which represents the early phase of IFN response (132–135). However, in primary human macrophages, stimulated by TLR2 ligands, IFNγ induces a strikingly different response. It reprograms metabolic pathways toward enhanced mitochondrial pathways and oxidative phosphorylation by inhibition of mTORC1 as well as MNK (136) (Figure 4). IFNγ treatment of patients with sepsis also enhances mitochondrial oxidative phosphorylation in peripheral blood mononuclear cells (137). mTORC1 and mTORC2 are well-known to control a wide array of metabolic pathways, including glycolysis, oxidative phosphorylation, and lipid metabolism (5, 138). Moreover, IFNγ suppresses the translation of repressors of inflammation, including HES1, HEY1, and IκBα, *via* mTORC1 in human macrophages (136, 139). The translational inhibition of these molecules promotes an inflammatory response and may contribute to the potent proinflammatory effects of this cytokine (140). Similarly, diminishing translation by blocking mTORC1 with rapamycin favors the translation of the more abundant proinflammatory cytokines such as IL-12 and blocks the translation of low abundant mRNAs such as IL-10 (5, 141). Mechanistically, IFNγ induces IDO1 expression (as explained above) by depleting intracellular tryptophan levels, and this suppresses mTORC1 (136). The amino acids leucine, arginine, as well as tryptophan are sensed by mTORC1 at the level of the lysosome. Only if sufficient amino acids are present, full mTORC1 activation by growth factors or PRR ligands occurs (142, 143). Additionally, IFNγ inhibits expression of the macrophage colony-stimulating factor receptor and interferes with the expression of SIRT1, a major deacetylase that influences energy metabolism and longevity (136, 144). Together, these data indicate that both the control of the cellular metabolism and mTORC1 activation by IFNγ may be central mediators of this pleiotropic proinflammatory molecule.

## Concluding Remarks

Based on recent and older studies, a theme is emerging, which shows that IFNs are potent modulators of basic cellular processes (Table 1). Viruses rewire the metabolism of the host cell to efficiently replicate and produce infectious particles. Therefore, interfering with distinct metabolic pathways seems to constitute one of the core antiviral properties of IFNs. In this context, we described that inhibition of FA and cholesterol synthesis as well as induction of NO are to date the best studied metabolic actions of IFNs. Future studies should focus on expanding the investigation of the influence of IFNs on the cellular energy metabolism including FAO. Moreover, IFN-mediated metabolic effects may be mediated by metabolic-derived protein and epigenetic modifications such as N-glycosylation, methylation, or acetylation (44, 145, 146).

**Table 1:** **Metabolic changes caused by interferons (IFNs)**.

Effects of IFNs	Cell type	Reference
Generally, IFNs cause a translational inhibition, but promote the transcription of IFN-stimulated genes		(1, 4, 10, 12, 130, 131–134)
↑Glucose uptake	mouse embryonic fibroblasts, human plasmacytoid dendritic cells (pDCs)	(31, 40)
↑Glycolysis	Splenic CD11c^+^ MHCII^+^ DCs *ex vivo*, macrophages, human squamous carcinoma cell line	(32, 34, 35, 43)
↑Aerobic glycolysis	Macrophages	(33)
↓Oxidative phosphorylation and adenosine triphosphate (ATP) production	Human squamous carcinoma cell line, mouse L929 or human Daudi cells, human CD4^+^ T cells *ex vivo*, pDCs	(35–37, 40)
↑Oxidative phosphorylation and ATP production	Primary human macrophages, peripheral blood mononuclear cells, pDCs, conventional DCs, keratinocytes, or memory T cells	(39, 135, 136)
↑Lipolysis	Adipocytes of mice *in vivo*	(41)
↑Itaconic acid	Alveolar macrophages	(47)
↑NO, reactive oxygen species	Macrophages, primary hepatocytes, macrophages	(30, 33, 113, 114)
↓Fatty acid and cholesterol synthesis	HeLa cells	(86)
↑25-hydroxycholesterol	Macrophages	(88)
↑Membrane rigidity	Daudi cells, L9292 cells, human monocytes, RSa, RSb, IF r and RD-114 cells, red blood cells of patients with hepatitis C infection	(91–95)
↓polyamine synthesis	Huh7 cells, BHK-21 cells	(103)
↑IDO1 expression, ↓of tryptophan	Macrophages, fibroblasts, pDCs, *Toxoplasma gondii, Salmonella enterica* serovar Typhi (*S*. Typhi), or *Chlamydia pneumoniae* infections	(119, 120, 123, 124)
↑AKT–mTOR complex 1, mTORC2, MAP kinase-interacting kinases, eukaryotic initiation factor 4E	Huh-7, Huh-7.5, 293T, MT-4, *STAT1^−^*^/^*^−^* Fib, reviewed in, MEFs	(131–134)
↓Translation of repressors of inflammation	Human macrophages (135, 138)	(135, 138)

However, some considerations need to be taken into account when studying metabolic processes. First, the metabolism of immortalized cell lines may be notably different from that of primary cells. Proliferating cell lines harbor mutations in pathways that regulate metabolic processes such as the mTOR pathway and already show a Warburg effect to allow infinite proliferation. Moreover, the composition of the cell culture medium profoundly affects the cellular metabolism. Culture media often contain nutrients that far exceeds the amounts observed in tissues and thus may mask the importance of individual metabolic pathways for specific immunologic functions. Finally, pharmacological inhibitors are instrumental in metabolic studies (147), but they may show off-target effects and should, therefore, be complemented with genetic studies to elucidate whether an observed metabolic shift is the cause or the consequence of a change in the cellular phenotype (148).

The expansion of our knowledge on immunometabolism and the role of IFNs suggest novel avenues for metabolic therapies. In this regard, it might be possible in the future to target specific pathways that are critical for viral replication, such as FAS or cholesterol synthesis. More generally, distinct immune cells may be more dependent on specific metabolic processes than others, and hence, more vulnerable to allow specific immunometabolic targeting of cells *in vivo* (149, 150). Moreover, the support of antiviral actions of IFNs by providing metabolites may also be possible. In conclusion, the reciprocal regulation of IFNs and metabolic processes advances our understanding of immunometabolism and may hold future surprises for our understanding of immunity in health and disease.

## Author Contributions

SF and TW conceived and wrote the manuscript.

## Conflict of Interest Statement

The authors declare that the research was conducted in the absence of any commercial or financial relationships that could be construed as a potential conflict of interest. The reviewers CD, CK and handling Editor declared their shared affiliation, and the handling Editor states that the process nevertheless met the standards of a fair and objective review.

## Abbreviations

2-DG, 2-deoxyglucose; 25-HC, 25-hydroxylcholesterol; ACC, acetyl coenzyme A carboxylase; Acetyl-CoA, acetyl coenzyme A; ACLY, ATP citrate lyase; ADP, adenosine diphosphate; ADV, adenovirus; αKG, α-ketoglutarate; AMPK, 5-adenosine monophosphate-activated protein kinase; ATP, adenosine triphosphate; cGAS, cyclic guanosine monophosphate-adenosine monophosphate synthase; ER, endoplasmic reticulum; F6P, fructose-6-phosphate; FA, fatty acid; FADH_2_, flavin adenine dinucleotide; FAO, fatty acid oxidation; FAS, fatty acid synthesis; G6P, glucose-6-phosphate; Glut4, glucose transporter 4; HBV, hepatitis B virus; HCMV, human cytomegalovirus; HCV, hepatitis C virus; Hif1α, hypoxia-inducible factor 1α; HIV, human immunodeficiency virus; HSV1, herpes simplex virus; H_2_O_2_, hydrogen peroxide; IDO1, indoleamine-2,3-dioxygenase 1; IFN, interferon; IFNAR1/2, interferon alpha and beta receptor subunit 1/2; IFNR, interferon receptor; IL-1, interleukin-1; IRG1, immune-responsive gene 1; NOS2, nitric oxide synthase 2; IRF, interferon-regulatory factor; IRG, immune-responsive gene; ISG, interferon stimulated gene; Kyn, kynurenine; LCMV, lymphocytic choriomeningitis virus; LPS, lipopolysaccharide; MDA5, melanoma differentiation antigen 5; MHV-68, murine gammaherpesvirus-68; MNK, mitogen-activated protein kinase (MAPK)-interacting kinase; mTOR, mammalian target of rapamycin; mTORC1/2, mTOR complex 1/2; NAD, nicotinamide adenine dinucleotide; NADP, nicotinamide adenine dinucleotide phosphate; NO, nitric oxide; NOS, nitric oxide synthase; OAA, oxaloacetate; OAS, 2′,5′-oligoadenylate synthetases; ONOO^-^, peroxynitrite; OXPHOS, oxidative phosphorylation or electrone transport chain; PAs, polyamines; pDC, plasmacytoid dendritic cell; PKR, double-stranded RNA-activated protein kinase; PRR, pattern recognition receptor; ROS, reactive oxygen species; SAT1, spermidine-spermine acetyltransferase; SFV, semliki forest virus; SREBP 1/2, sterol regulatory binding protein 1/2; STAT, signal transducer and activator of transcription; STING, stimulator of interferon gene; SOD1, superoxide dismutase 1; TCA, tricarboxylic acid; TLR, Toll-like receptors; Treg, regulatory T cell; Trp, tryptophan; TYK2, tyrosine kinase 2; UDP-GlcNAc, uridine diphosphate-*N*-acetylglucosamine; VSV, vesicular stomatitis virus; VV, vaccinia virus; WNV, West Nile virus.
